# Diseases of the Blood

**Published:** 1905-01-28

**Authors:** 


(*To be continued.*)

### DISEASES OF THE BLOOD.

**Disorders of the Blood in Early Life.**—In the Goulstonian lectures Hutchison[1] gave an exhaustive account of the present state of knowledge of the disorders of the blood in early life. The infant starts life with an excess of red corpuscles in the blood—about 5,500,000 per cub. mm.—but this passes off by the end of the second week, after which time the number is about normal. The cause of this excess is probably a sudden increase in the activity of the blood-forming organs rendered necessary by a change of the conditions of existence. The proportion of hæmoglobin at birth is in proportion to the richness of the blood in red corpuscles; but it soon sinks, and from the age of about six months to the beginning of the sixth year is below the adult standard. The blood of the young child is thus comparatively poor in hæmoglobin. The cause of this chlorotic condition is not quite clear. There are two stages of red blood formation during intra-uterine life: an embryonic stage, in which the blood vessels, and perhaps also the liver, are the sites of formation, and the fœtal stage proper, in which red cells are formed in the liver, spleen, and bone-marrow. The two stages pass into one another about the fourth month of intra-uterine life. As birth approaches the liver and spleen cease to form red cells, and after birth they are only developed in the red marrow, which during childhood occupies the whole of the interior of bones. As puberty approaches the shafts of the bones are filled with fat, and the red marrow becomes confined to the ends of the long bones and the whole of the flat or spongy bones. A child thus lives up to the limits of his blood-forming capacity, and thus bears hæmorrhage badly and easily becomes anæmic. The white cells of the blood can be divided into granular and non-granular. The former includes the polynuclear neutrophile and eosinophile leucocytes, the latter the lymphocytes, large lymphocytes, large mononuclears, and transitionals. During infancy there is a polynuclear leucocytosis, most marked just after birth, followed by a slow rise in the non-granular cells, which reaches its maximum about the sixth month, but which persists in a less pronounced form throughout the whole period of infancy. The significance of the initial polynuclear leucocytosis is not understood. As regards the latter point it would appear to indicate a great activity of the lymphoid tissue throughout the body, and the nearer the organism stands to the fœtus the more easily can a return to fœtal conditions take place, and the lymphoid cells of the marrow assume the function of producing lymphocytes, and those of adenoid tissue that of producing granular cells. The great activity of the lymphoid tissue during infancy is probably due to their playing some part in nutrition, possibly in carrying fat. In infancy there is thus a high absolute number of non-granular leucocytes and a normal number of red cells relatively poor in hæmoglobin. Congenital abnormalities of the blood may occur in the blood considered as a tissue, in the total quantity of the blood or one of its cellular constituents, and in its chemical composition. Thus in the condition known as infantilism there is often the marked excess of non-granular cells characteristic of infancy, and which normally disappears at or before the sixth year. Again, there are infants who are markedly anæmic from birth, quite independent of such causes as congenital syphilis. This anæmia in some cases may affect chiefly the hæmoglobin, suggesting that the child has come into the world with a defective supply of iron in its liver. When this is the case steady improvement follows the administration of iron. In other cases no such improvement follows, and these may be due to a congenital defect in the red marrow and a consequent insufficient formation of red cells. In congenital cyanosis there is an excess of red cells and of hæmoglobin, and examination of such cases after death shows an undue activity of the blood-forming organs and a persistence of the fœtal method of intra-capillary blood formation. Cases are occasionally met with in infancy of anæmia of a markedly chlorotic type associated with the greenish complexion, hæmic murmurs, and other well-recognised symptoms of the disease as met with in girls. This is not surprising, for the blood of infancy is normally poor in hæmoglobin, and the presence of any factor which makes its production more difficult will lead to the appearance of this type, *e.g.* too long suckling by the mother—for milk is notably deficient in iron. Pernicious anæmia is exceptionally rare in childhood—which is surprising considering the vulnerability of the blood in childhood, the frequency of oral sepsis, and the ease with which the fœtal mode of blood formation is resumed. Secondary anæmia is common: the hæmoglobin seems to suffer first, and then, if the injurious influence become greater, the number of the corpuscles. In such circumstances irregular varieties of red cells (poikilocytosis) and nucleated forms very often make their appearance, this being the characteristic of secondary anæmia in childhood as compared with its features in later life. The red marrow can only respond by more rapid production of immature forms; it cannot increase in amount, the whole of the cancellous tissue being normally full of it. In secondary anæmia, particularly when dependent on chronic gastro-intestinal disorders, lymphocytosis frequently occurs—possibly owing to over-production, to make up for the increased difficulty in food-absorption. Malnutrition, due to poverty of food in iron and in proteid, is very apt to lead to anæmia in childhood. Acute infective diseases cause anæmia probably by an increased destruction of the blood. Thus rheumatism may cause a fall in the number of the red corpuscles of as much as 1,000,000 per cub. mm. in four or five days. Other similarly acting causes are diphtheria, syphilis, tuberculosis, chronic diarrhœa. In infantile scurvy the blood exhibits merely a varying degree of that secondary anæmia of rather chlorotic type which is so common in rickets and other conditions of impaired nutrition. Nor does examination of the marrow exhibit any characteristic changes. In the majority of cases the child is being fed on a diet which is deficient in fresh constituents, particularly in fresh milk. Two theories have been put forward: that there is present in the food some constituent which it should not contain (*e.g.* ptomaines), and that there is absent from it one of its normal constituents. Now the addition of sterilised fruit juice to the food will affect a removal of the symptoms. The fault is, therefore, one of omission, and not one of commission. Dialysis of such fruit juice showed that the uncrystallisable or colloid constituents were devoid of any curative effect, whilst the crystalline constituents cured. On the other hand, potassium tartrate, potassium citrate, and potassium malate (the chief constituents of fruit juice) do not give anything like such good results as are obtained from the natural juice. One is entitled to conclude, however, that the defect in the blood in scurvy is a poverty in vegetable salts. It may be remarked that the hæmorrhages cannot be due to a diminished coagulability of the blood, for this is normal. A diminution of the vegetable salts of potash would diminish its alkalinity, and this diminished alkalinity of the blood is present in adult scurvy. The stoppage of a tinned food, which often leads to the happiest results in scurvy, may simply act by diminishing the supply of the more acid salts to the blood.

[1] Hutchinson, Lancet, May 7, 14, and 21.

*(To be continued.)*

